# Can Amplicon Sequencing Be Replaced by Metagenomics for Biodiversity Inventories?

**DOI:** 10.1111/1755-0998.70047

**Published:** 2025-09-25

**Authors:** Lucas Elliott, Eric Coissac

**Affiliations:** ^1^ UiT—The Arctic University of Norway Tromsø Norway; ^2^ Univ. Grenoble Alpes, Univ. Savoie Mont Blanc, CNRS, LECA Grenoble France

Biodiversity assessments are a critical part of ecological monitoring, food systems management, and many areas of research. Traditionally, recording which taxa are present in an area has been accomplished by resource‐intensive surveys and the morphological identification of bulk samples by taxonomic experts. The advent of DNA metabarcoding has allowed many of these barriers to be circumvented by amplifying and sequencing taxon‐specific DNA loci to create an inventory of what organisms are present in a sample. However, this method is limited to a small fraction of DNA present in a sample and is biased towards over‐ and under‐representing certain organisms based on their genomic content. With continually decreasing sequencing and computational costs, metagenomic analysis of the entire DNA content of a sample aims to capture a more complete picture of an area's biodiversity. Callens et al. (2025) provide a direct comparison of metabarcoding and metagenomic analysis on morphologically identified macrobenthos bulk samples and detail a strategy for expanding the use of metagenomics in bulk sample characterisation. In their case study, metagenomics enabled the composition and biomass of the organisms in the samples to be reconstructed with greater accuracy. Contrary to common belief, this was achieved with a similar level of sequencing effort to that required for metabarcoding. Can this approach be generalised to any biodiversity inventory with the same success?

The DNA‐based taxonomic annotation of environmental samples (eDNA) and bulk samples to characterise the biodiversity of an area has seen a rapid growth of implementation in the past decades. In addition to having a lower resource cost than surveys, DNA‐based methods can detect species that are difficult to morphologically identify or observe in the wild. Previous comparisons between the DNA‐based metabarcoding and metagenomic workflows have documented both largely overlapping taxonomic detections (Courtin et al. 2022) as well as minimally overlapping datasets with inverse alpha diversity patterns (Hollman et al. 2025).

Metabarcoding has been the most established and widely implemented of the two workflows, but metagenomics offers several advantages and challenges by comparison. By sequencing the entire DNA content of a sample with metagenomics, all organisms can potentially be identified and quantified instead of limiting the detection to a specific taxonomic group with metabarcoding primers. However, without amplification, DNA from taxa of interest is at risk of being swamped by organisms with a higher total biomass or being preferentially captured in a sample, leading to false negatives (Zimmermann et al. 2023). In environmental samples, non‐microbial DNA typically represents less than a third of the total DNA (Eisenhofer et al. 2024), and sometimes less than 10%.

By not focusing on taxon‐specific DNA loci, metagenomics allows for the study of genome‐wide regions, opening the doors to a plethora of possible phylogenetic and functional analyses (Gelabert et al. 2021). However, due to the size of eukaryotic genomes and the extent to which conserved regions are shared across taxa, only a small portion of plant and metazoan genomes is taxonomically informative at the species level, resulting in many metagenomic studies limiting taxonomic assignment to the genus level (e.g., Wang et al. 2021; Elliott et al. 2025). By contrast, metabarcoding primers can achieve species‐level resolution for over 50% of detected taxa (Garcés‐Pastor et al. 2022). Metagenomic workflows require increased sequencing and computational resources due to non‐informative DNA fragments combined with the overrepresentation of bacterial DNA in environmental samples, leading to a greater overall footprint for metagenomic studies. However, the resulting large datasets can be more readily reanalyzed and repurposed for future studies.

On the quantitative aspect, Callens et al. (2025) report a stronger correlation between relative read abundance and biomass from metagenomics compared to metabarcoding. However, the COI gene used for metabarcoding in this study aims to amplify a wide diversity of taxa at the cost of many mismatched primer binding sites (Deagle et al. 2014). Other, more taxonomically limited barcoding regions have been shown to produce a stronger correlation between read count and biomass (Elbrecht et al. 2016). Given the taxonomic diversity and high endogenous DNA content of macrobenthos samples, Callens et al. (2025) demonstrate how metagenomics is a more appropriate workflow in this context contingent on a complete and equally representative database being available. While metagenomics avoids the biasing effect of large numbers of PCR amplification cycles required by metabarcoding, it cannot be considered a completely unbiased method as various factors such as guanine‐cytosine content are known to influence the final DNA read counts of organisms (Browne et al. 2020).

Bulk samples have a high concentration of fresh, endogenous DNA, which can be detected at low sequencing depth by metagenomics (Callens et al. 2025), whereas environmental DNA samples are dominated by microbes and have a much higher DNA complexity, requiring orders of magnitude more sequencing. Due partially to this complexity of metagenomic datasets, false positive taxon identifications are often a risk, with many tools reporting a baseline rate and recommending filtering at a percentage of the total read count (Pedersen et al. 2016). Callens et al. (2025) calculate this threshold to be 0.2% of the dataset, noting that even with a high percentage of fresh, endogenous DNA, the entire reference database appears as present in every sample at read counts below this percentage. This study was performed with a small database of 26 organisms, whereas bulk samples, and especially environmental samples, can contain orders of magnitude more diversity.

One of the greatest obstacles for metagenomic analysis is the lack of high‐quality reference material where much of this diversity is not represented. Genome skimming or low‐coverage whole genome sequencing offers an efficient method for expanding reference databases (Alsos et al. 2020; Lavergne et al. 2025). Previous studies incorporating even partially assembled versions of this data show a massive increase in the number of DNA reads that can be taxonomically annotated (Wang et al. 2021). To take full advantage of the information contained in the genome skims, Callens et al. (2025) use a k‐mer‐based approach on the unassembled genome skims as reference material, demonstrating that at even 1× coverage, a majority of reads from that species can be classified. The upscaling of unassembled genome skims used as a reference database has been shown to be computationally challenging, with programs such as kraken2 (Wood et al. 2019), but can be implemented with probabilistic data structures (Elliott et al. 2025).

Ultimately, neither metagenomic nor metabarcoding methods can be definitively preferred over the other for all analyses. As always, the choice between the two is highly dependent on the research question, available funds, and sample composition and origin. Understanding the strengths and limitations of both workflows is critical to interpreting their results. Callens et al. (2025) demonstrate the utility of metagenomics compared to metabarcoding when applied to biodiversity assessments of bulk samples. Expanding reference databases with low coverage genome sequencing while simultaneously developing computational tools for managing this large amount of data will continually expand the value of metagenomics in the future. Nevertheless, as scientists, it is important to bear in mind that a measure of reality is not reality itself. We must understand the limitations of our tools and select the most appropriate one for each task, as the tool that is most effective for one task may be the least effective for another.

## Author Contributions

L.E. and E.C. conceived and wrote the manuscript.

## Conflicts of Interest

The authors declare no conflicts of interest.

## Data Availability

The authors have nothing to report.
